# Challenges of conducting a randomised placebo-controlled trial of spinal surgery: the SUcceSS trial of lumbar spine decompression

**DOI:** 10.1186/s13063-023-07772-5

**Published:** 2023-12-06

**Authors:** Emma Kwan-Yee Ho, Ralph Jasper Mobbs, James Montague van Gelder, Ian Andrew Harris, Gavin Davis, Ralph Stanford, David John Beard, Christopher Gerard Maher, Joanna Prior, Michael Knox, David Barrett Anderson, Rachelle Buchbinder, Manuela Loureiro Ferreira

**Affiliations:** 1 The University of Sydney, Sydney Musculoskeletal Health, Charles Perkins Centre, Faculty of Medicine and Health, School of Health Sciences, Sydney, NSW 2050 Australia; 2 The University of Sydney, Sydney Musculoskeletal Health and the Kolling Institute, Faculty of Medicine and Health, School of Health Sciences, Northern Sydney Local Health District, St Leonards, NSW 2065 Australia; 3 NeuroSpine Surgery Reserach Group (NSURG), Sydney, NSW 2031 Australia; 4 Prince of Wales Hospital, Barker Street, Randwick, NSW 2031 Australia; 5 School of Medicine, University of New South Wales, Sydney, Australia; 6 Department of Neurosurgery, Concord Repatriation General Hospital, Concord, NSW 2139 Australia; 7 Sydney Spine Institute, Burwood, NSW 2134 Australia; 8 South West Sydney Clinical Campuses, School of Clinical Medicine, UNSW Medicine & Health, University of New South Wales, Sydney, NSW 2170 Australia; 9 Neurosurgery, Cabrini & Austin Hospitals; and School of Public Health and Preventive Medicine, Monash University, Melbourne, VIC 3800 Australia; 10 NHMRC CTC, Faculty of Medicine and Health, University of Sydney, Sydney, Australia; 11 The University of Sydney, Sydney Musculoskeletal Health, Sydney, NSW 2050 Australia; 12 The University of Sydney, School of Health Sciences, Faculty of Medicine and Health, Sydney, NSW 2050 Australia; 13 School of Public Health and Preventive Medicine, Monash University, Melbourne, VIC 3800 Australia; 14 Musculoskeletal Health and Wiser Health Care Units, School of Public Health and Preventive Medicine, Monash University, Melbourne, VIC 3004 Australia

## Abstract

Although placebo-controlled trials are considered the gold standard for evaluating the efficacy of healthcare interventions, they can be perceived to be controversial and challenging to conduct for surgical treatments. The SUcceSS trial is the first placebo-controlled trial of lumbar decompression surgery for symptomatic lumbar canal stenosis. The SUcceSS trial has experienced common issues affecting the implementation of randomised placebo-controlled surgery trials, accentuated by the COVID-19 pandemic. Using the SUcceSS trial as an example, we discuss key challenges and mitigation strategies specific to the conduct of a randomised placebo-controlled surgical trial. Overall, the key lessons learned were (i) involving key stakeholders early and throughout the trial design phase may increase clinician and patient willingness to participate in a placebo-controlled trial of surgical interventions, (ii) additional resources (e.g. budget, staff time) are likely required to successfully operationalise trials of this nature, (iii) the level of placebo fidelity, timing of randomisation relative to intervention delivery, and nuances of the surgical procedure under investigation should be considered carefully. Findings are based on one example of a placebo-controlled surgical trial; however, researchers may benefit from employing or building from the strategies described and lessons learned when designing or implementing future trials of this nature.

## Introduction

Randomised controlled trials (RCTs) are considered the gold standard for evaluating the efficacy or comparative effectiveness of health interventions. Through the process of random allocation, RCTs aim to achieve an equal distribution of prognostic and confounding factors across treatment groups to produce an unbiased estimate of the treatment effect. While high-quality RCTs comparing surgical interventions to non-surgical interventions provide evidence for overall treatment efficacy, they do not account for specific biases associated with placebo effects or knowledge of treatment allocation. Particularly for surgical interventions, placebo effects have been shown to have substantial magnitude and duration [1]. Further, when group allocation is not blinded, strong patient preference for a specific treatment combined with knowledge of treatment allocation can potentially lead to consistent variations in the way outcomes are reported amongst intervention groups. Lack of blinded treatment allocation can also result in high treatment crossover rates which may, in turn, introduce performance bias and underestimate true treatment effects [2, 3].

Bias within RCTs can be reduced by blinding participants and observers by using a placebo control group. Placebo-controlled trials are designed to account for the non-specific effects of a health intervention (i.e. placebo response), by mimicking the content and delivery of an intervention minus the proposed active component [4–6]. In the context of surgical trials, this may include possible non-specific placebo effects associated with undergoing a surgical procedure, including consultation, anaesthesia, and/or accompanying post-surgical rehabilitation. This can be achieved through blinding. Compared with trials of surgical versus non-surgical care, where blinding of participants is difficult to achieve due to marked observable differences between intervention groups, placebo-controlled surgery trials may allow for more reliable blinding of participants and outcome assessors [7] to protect against treatment allocation bias. In turn, blinding may also help reduce treatment crossovers, which are known to introduce performance bias [2, 3]. As a result, placebo-controlled surgical trials are useful for minimising bias in the assessment of the treatment effects of a proposed surgical procedure. Nevertheless, despite evidence suggesting that randomised placebo-controlled surgery trials are feasible and safe [4, 8], their use can be perceived to be controversial.

The SUrgery for Spinal Stenosis (SUcceSS) trial is an ongoing randomised placebo-controlled trial evaluating the efficacy, safety, and cost-effectiveness of decompression surgery in people with symptomatic lumbar canal stenosis who have not responded to non-operative care. Participants are being randomised to either lumbar decompression surgery or placebo surgery, with participants, assessors, and clinicians other than the operating surgeon, being blinded to group allocation. Further details of the SUcceSS trial, including the rationale and methods for data collection, have been published in the protocol [5]. The SUcceSS trial has experienced common issues affecting implementation of randomised placebo-controlled surgery trials, accentuated by the COVID-19 pandemic. As the first trial comparing decompressive surgery with placebo surgery in people with lumbar canal stenosis, additional resources were allocated to establishing feasibility due to a paucity of literature guiding trial design. Using the SUcceSS trial as an example, we discuss key challenges and strategies which are specific to the conduct of randomised placebo-controlled surgical trials. We focus on issues relating to feasibility, participant safety, and design, as well as unanticipated challenges such as the COVID-19 pandemic. We conclude with key lessons learned.

### Feasibility

To ensure the feasibility of trial completion, placebo-controlled trials of surgical interventions require additional resources to ensure sufficient clinician buy-in, as well as full consideration of patient perspectives about a proposed placebo intervention during the trial design phase. Consideration of these factors is especially important for trials of placebo-controlled interventions which are being evaluated for the first time in clinical trial.

#### Gaining buy-in from surgeons

In placebo-controlled trials of surgery, particularly in trials evaluating surgical procedures which are considered standard care, gaining buy-in from clinicians including surgeons can be challenging and significantly impact recruitment. Surgeons must be willing to: explore and satisfy their own position of clinical equipoise regarding the procedure under investigation; familiarise themselves with the rationale, conduct, and implications of placebo-control methodology [9]; and act as recruiting surgeons and encourage participation from their patients and surgical colleagues. Whilst buy-in from most of the clinical community is desirable, it is not often the case in studies such as placebo trials of surgery.

In the SUcceSS trial, prior to implementation, several consultations were held with groups of potential recruiting surgeons to ascertain whether enough surgeons were willing and able to recruit the intended sample size within the trial timeline. From these consultations, a craft group of surgeons who were committed to overseeing successful trial completion was formed. Unsurprisingly, throughout various stages of trial implementation, resistance from less supportive and unsupportive surgeons has been experienced, which has impacted recruitment and placed additional onus on the participating surgeons. Main barriers cited by unsupportive surgeons have included the perception that a placebo-controlled trial for lumbar decompression is unnecessary due to anecdotal evidence that the procedure is effective; discomfort with challenging a routinely used surgical procedure; and perceived difficulties with operationalising the trial (e.g. introducing the trial to participants, logistics of performing the placebo surgery, challenges with maintaining blinding). Geographical location and logistics of billing have not been cited as reasons affecting clinician buy-in.

Nevertheless, several strategies have been implemented which have successfully increased clinician buy-in for the trial. For example, extensive efforts have been made to foster strong partnerships with “champion” recruiting surgeons and research positive hospitals, leverage the clinical networks of recruiting surgeons and trial investigators to identify additional surgeons and recruitment sites, and outreach to orthopaedic and neurosurgeons by presenting information on the trial, and opportunities for collaboration, at national and international conferences. These parallel strategies have resulted in an expansion from 10 supportive surgeons across 6 recruitment sites during early trial implementation, to 17 surgeons across 13 sites to date. Typically, surgeons who are supportive of the trial share awareness that most evidence for surgical interventions is based on retrospective case series or professional opinion [10] as opposed to RCTs [11], and there is currently no high-quality RCT evidence supporting the benefits of lumbar decompression for spinal stenosis. Further, many of the supportive surgeons are also involved in research.

#### Consideration of patient perspectives regarding the proposed placebo intervention

Early consideration of patient perspectives regarding a proposed placebo treatment is crucial to ascertain feasibility of recruitment (i.e. willingness to participate in a trial involving the proposed placebo intervention) and ensure patient concerns are adequately addressed in trial information and consent materials. Prior to implementation of the SUcceSS trial, we invited 68 Australians with lumbar stenosis and indication for lumbar decompression surgery to complete an online survey to determine patient willingness to participate in a placebo-controlled trial of decompression surgery [12]. Sixty-three (91%) participants responded to the survey and provided information on (i) their willingness to participate in a trial involving surgery for lumbar spinal stenosis, (ii) their willingness to participate in a trial of surgery for lumbar spinal stenosis which involves random assignment to either surgery or placebo surgery, and (iii) whether or not, if they were randomised to the placebo surgery group, they would be willing to wait for 6 months after their index surgery before receiving traditional decompression surgery. Participants who were unwilling to participate in a trial of this nature were asked to provide reasons for non-consent, via series of open-ended questions.

Results of the survey established that almost all participants (59 of 63 participants, 95%) acknowledged the need to conduct clinical trials to evaluate treatments with unknown efficacy, with 16% (*n* = 10) indicating their willingness to participate in a placebo-controlled trial of lumbar decompression surgery. The main barriers towards participation were lack of information about the procedure (lumbar decompression surgery), lack of reassurance of a positive outcome with participation (even if they receive placebo), as well as concerns about the risks and benefits of placebo surgery (e.g. infection). These results informed the design of the SUcceSS trial, including but not limited to the development of a lay explanation of the trial rationale and procedures; trial procedures before, during and after surgery (e.g. nature of information discussed during trial introduction, frequency of follow-up care with the surgical team, post-operative communication between the trial team and the patient’s general practitioner, as needed; frequency and medium used for follow-up data collection); and the production of all patient-facing documents such as the information sheet, consent form, and advertising materials in different formats (e.g. leaflet, poster, infographic). Of note, after the initial drafts of patient-facing documents were prepared (based on the survey results) these materials underwent further review by a consumer feedback panel to ensure acceptability and readability by the target audience. Concurrently, a standard script to assist participating surgeons and research staff to introduce the trial to patients was developed which included these iterations. The script was created in collaboration with clinicians, consumers, and an ethicist.

During trial implementation, mixed patient perspectives on the trial and the placebo surgery group have emerged. The most common reason that potential patients have declined participation during trial introduction is due to strong beliefs that lumbar decompression surgery is the most effective treatment for their symptoms. Some potential patients, particularly older patients, have also expressed concerns regarding general risks of anaesthesia and surgery and declined participation (randomisation) to avoid the possibility of requiring further surgery — a risk which many patients perceive to be higher in the placebo surgery group. On the contrary, several factors have been observed amongst patients who have agreed to participate in the trial. For example, a strong endorsement of participation from a patient’s treating surgeon, general practitioner, and/or family members at the outset of introduction to the trial, patients with pronounced altruistic inclinations or those with greater health literacy surrounding the importance of placebo RCTs. One or more of these factors, combined with the possibility of an accelerated timeline for surgery, appear to have positively impacted a patient’s willingness to participate in the trial.

It is worth noting that whilst the feasibility survey revealed that only 16% of respondents were willing to participate in a placebo-controlled trial of lumbar decompression surgery, patient willingness was not re-assessed after extensive efforts were made to refine the patient facing materials based on patient-reported concerns and feedback. It is highly likely that with these critical changes, re-assessment would have revealed a higher percentage of patients willing to participate in the proposed trial.

### Participant safety

Despite evidence showing that placebo-controlled trials of surgery pose minimal risk to patients [4, 8], additional measures are required to ensure participants are fully informed and reassured of all possible risks of the placebo intervention. This can include creating additional resources to educate and reassure potential participants of the contents and possible risks of the placebo intervention, enhancing trial monitoring procedures, and consulting with appropriate external stakeholders to minimise the risk of therapeutic misconception.

#### Educating and reassuring potential participants regarding the placebo intervention

Ensuring that patient concerns about a proposed placebo intervention are adequately addressed in during the trial information session and in the written trial consent materials is crucial to ensure informed consent and participant safety. In the SUcceSS trial, results of the feasibility survey to determine patient willingness to participate in a placebo-controlled trial of decompression surgery revealed that concerns about the risks of placebo surgery were a main barrier for participation. As a result, we allocated additional resources to develop a recruitment video (https://youtu.be/cXJgso1BSJE) to assist surgeons and research staff with introducing the trial to potential participants. The intention of the video is to ensure that potential participants are fully aware of all procedures and possible risks involved with taking part in the trial. In the video, a trial surgeon who is also an investigator discusses information on the following topics: (i) ‘Why is this study being done?’, (ii) ‘Who is being invited into the study?’, (iii) ‘What will the study involve?’, (iv) ‘Who you can contact in case you have any questions?’. To ensure potential participants fully understand the differences between the intervention groups, the video includes an animation of the procedures which occur in the surgical decompression (i.e. removal of bone and/or ligament) and placebo surgery (no removal of bone and/or ligament). The contents of the video were approved by the reviewing ethics committee. Most commonly, the research team shares the video link with all potential participants after the trial surgeon has already introduced the trial and prior to signing of the consent form. Anecdotally, participants have provided informal feedback that the video, particularly the animation, assisted with visualisation and understanding of the physical differences in the operative procedures across both trial arms. This is consistent with previous evidence suggesting that video animations may positively enhance patient understanding and attitudes about participation in research [13]; however, the effectiveness of animated consent materials specifically in the context of placebo-controlled surgical trials remains unknown.

Furthermore, during trial introduction, intentional efforts are made to reassure potential participants that the potential risks in the placebo surgical procedure are no greater than those in the surgical decompression arm [4]. This is because the placebo procedure excludes the process of removing bone and/or ligaments, which is associated with most adverse events and complications of surgical decompression (e.g. dural tear, cerebrospinal fluid leak) [14, 15]. Further, revision surgery is a common complication of lumbar decompression surgery [16], therefore, the risk of requiring further surgery can occur in both arms of the trial.

Ultimately, it is unclear whether the consent video and targeted efforts to reassure patients regarding the potential risks of the placebo surgery have directly impacted patient willingness to participate in the SUcceSS trial. This is because other contextual factors such as statewide bans on non-urgent elective surgeries and lockdown restrictions impacting patient capacity to attend medical appointments have significantly interfered with the rate of patient identification and randomisation (i.e. performance of the trial operation). Available literature suggests that enhanced consent (i.e. involving audiovisual materials) may help increase patient knowledge and satisfaction with the consent process [17]; although, the effect of animated consent material on participant willingness to enrol in placebo-controlled surgical trials remains unknown and is undergoing evaluation [13].

#### Enhanced trial monitoring procedures

As the first placebo-controlled trial of lumbar decompression surgery for symptomatic lumbar canal stenosis, the SUcceSS trial was considered as high-risk by the trial sponsor. As a result, enhanced trial monitoring measures were implemented to ensure the sponsor remains well informed of any potential risks to trial participants. Aside from increasing the frequency of sponsor reports from annually to quarterly, trial adverse event reports are reviewed monthly by an independent trial medical monitor to determine causality, severity, and need for clinical escalation. Every 3 months, participants are contacted by phone to gather information about any unfavourable incidents which may have occurred. The decision to perform follow-up data collection over the phone, instead of via other media (e.g. online surveys, face-to-face visits), was intentional. Phone calls were considered preferable given the demographics of the trial participants (e.g. aged over 50 years), convenience for participants and particularly for those travelling from rural areas, and higher risks associated with surgical interventions compared with non-surgical care. Further, any unfavourable incidents considered to be serious are reported to the sponsor within 24 h of notification, and the central trial management team will follow up with the participant until the incident is resolved. After 3 years of enhanced monitoring procedures, the sponsor has endorsed a reduction in the frequency of sponsor reports, from quarterly to annually, due to sponsor confidence in the safety of participants in the trial. Monthly review of adverse event reports and 3-monthly follow-up with participants regarding unfavourable incidents continue to occur.

#### Consulting with external stakeholders to minimise the risk of therapeutic misconception

Therapeutic misconception occurs when participants systematically misunderstand the purpose of research procedures (e.g. randomisation, placebo interventions) and incorrectly consider these to be included for their direct benefit [18]. Therapeutic misconception can therefore undermine informed consent. In placebo-controlled surgery trials, the risk of therapeutic misconception increases when researchers fail to disclose possible differences in the risk–benefit ratio of receiving a surgical placebo, versus the surgical intervention under investigation which is typically considered standard care. Consultation with appropriate external stakeholders (i.e. ethicist, independent surgeons) may be a useful strategy for minimising the risk of therapeutic misconception. The SUcceSS trial investigators met with an ethicist early in the trial design phase to review the trial documents (i.e. trial protocol, participant information sheet and consent form, and advertising materials), to ensure that all trial procedures and potential risks were appropriately disclosed to participants prior to consent, and use of medical jargon was limited. The ethicist also provided guidance on ensuring adequate patient comprehension of the likely (absence of) benefit from placebo allocation, as well as the appropriate timing to disclose the potential benefits of participation in the trial. That is, potential benefits (i.e. the index surgery would be performed at no cost to the participant) should only be mentioned to the patient following a discussion of the trial procedures and only upon patient request for further information. Furthermore, given the novelty of the SUcceSS trial design, it was advantageous to have an expert (ethicist) who could liaise with the reviewing ethics committee during the approval process. Based on advice from the ethicist, the SUcceSS trial investigators also sought feedback from independent surgeons, who contributed further expert advice on the therapeutic area and practicalities of operationalising the trial with patients in clinical practice.

### Design considerations

To ensure the validity of well-designed surgical RCTs that are placebo-controlled, it is crucial to meticulously select the most appropriate level of placebo fidelity, carefully time the point of randomisation relative to intervention delivery, and contemplate strategies to sustain blinding. Efforts to sustain blinding have concurrent benefits for reducing treatment crossovers, which are known to introduce performance bias and possibly underestimate true treatment effects [2, 3]. Neglecting these aspects could potentially undermine the integrity of the trial.

#### Placebo fidelity

In randomised placebo-controlled surgical trials, placebo fidelity describes the extent to which the index placebo surgery mimics the contents and attributes of the complete surgical intervention [19]. Level of fidelity ranges from minimal, low, to high, where minimal fidelity refers to placebo interventions involving minimal to no attributes of the procedure under investigation, whilst low fidelity refers to placebo interventions resembling few attributes of the procedure under investigation [19]. The appropriate level of fidelity may differ depending on the specific surgical procedure under investigation, ethical implications (e.g. risk–benefit ratio of a low versus high-fidelity placebo), logistics of blinding, and patient and surgeon acceptance (i.e. likelihood of participating) of a placebo surgical trial [19].

The SUcceSS trial is an example of a high-fidelity placebo-controlled surgical trial, whereby all contents and attributes of the placebo surgery are identical to the surgical decompression procedure, except for the presumed active or essential component. That is, all participants undergo identical pre-operative (i.e. clinical examination), peri-operative (i.e. anaesthetic protocols, skin incision, muscular dissection, and duration of anaesthetic), and post-operative care (i.e. standard follow-up care). However, the surgical decompression procedure involves the removal of bone(s) and/or ligament(s) at the spinal level(s) implicated in the patient’s presentation. Removal of bone and/or ligament to increase canal volume and reduce neural compression does not occur in the placebo surgery group. In the SUcceSS trial, a high-fidelity placebo control was selected based on a priority for selecting a control group which could best account for the non-specific effects of the index procedure. The decision was made with full consideration of the potential risks to patients and was further informed by evidence suggesting that high-fidelity placebo controls may better maximise blinding and reduce crossovers and dropouts compared with minimal-fidelity placebo controls [19].

#### Timing of randomisation relative to intervention delivery

In placebo-controlled trials of surgery, timing of randomisation relative to intervention delivery is critical for several reasons. The primary purpose is to minimise the risk of participants and assessors becoming accidentally unblinded to group allocation. Timing of randomisation also has other functions including reducing the chance of participant withdrawals between randomisation and the intervention (i.e. in the event where patients are no longer suitable for surgery due to unrelated or unexpected health complications) and ensuring that the surgical team approach all cases in an identical fashion, up to the time of randomisation. In the SUcceSS trial, disclosure of group allocation occurs mid-surgery when the participant’s surgical exposure has been performed. Immediately after skin incision and muscle retraction to expose the spine, a theatre clinician trained in the trial protocol (e.g. surgical assistant, anaesthetist) dials an interactive voice randomisation service (IVRS). The IVRS automatically discloses the participant’s group allocation to the theatre clinician over the phone. Once group allocation is revealed, for participants allocated to surgical decompression, the operating surgeon will continue with removal of bone and/or ligament. For participants allocated to placebo surgery, the surgeon will not progress any further with bone and/or ligament removal. Instead, the surgeon will proceed to close the wound after an equivalent time period that would be anticipated in the decompression allocation has elapsed. IVRS was purposely selected as the mechanism for randomisation, as the system is automatic and operates 24 h per day for 7 days per week, accommodating for sudden changes in operation times and enabling randomisation to occur immediately mid-surgery, with minimal disruption to patient care.

#### Maintenance of blinding and minimisation of treatment crossovers

Maintaining blinding in placebo-controlled trials of surgery is a vital but challenging process. In the SUcceSS trial, only the operating team (e.g. index surgeon, anaesthetists, surgical trainee, essential theatre nurse/s) and the clinical trial co-ordinator are unblinded to group allocation. All other stakeholders, including the participant, outcome assessors, and trial investigators, remain blinded for the entire study duration. There are two scenarios where blinded stakeholders become aware of a participant’s group allocation: planned unblinding, which occurs when a participant actively requests disclosure of their group allocation, which is permissible and must first be approved by the trial chief investigator, and accidental unblinding, which refers to all other scenarios where a participant incidentally becomes unblinded to their group allocation.

To elaborate further on accidental unblinding, there are numerous avenues in which patients, investigators, or assessors can become accidentally become aware of group allocation during the implementation of placebo-controlled surgical trials, especially in trials with longer follow-up. During the protocol design phase, it is essential to identify potential sources of accidental unblinding and proactively embed risk mitigation strategies to best protect data integrity. Iterative strategies should also be employed if newly discovered sources of accidental unblinding are identified during trial implementation.

In Tables 1 and 2, we describe several sources of accidental unblinding identified for the SUcceSS trial during the operative and post-operative phases, and the accompanying risk management strategies employed to overcome them. Overall, the main approach we recommend for placebo-controlled surgical trials is to: select an appropriate time point for randomisation relative to intervention delivery (i.e. operation); strategically restrict the number and type of trial personnel who are unblinded to group allocation (i.e. only the operating team and clinical trial co-ordinator); minimise interactions between unblinded personnel and trial participants; and ensure that participants and clinicians involved in their care are well-informed about trial procedures.

**Table 1:** Potential sources of accidental unblinding and risk mitigation strategies: operative phase

Potential source of accidental unblinding	Risk mitigation strategy
**Theatre times:** Marked differences in theatre time, between participants receiving surgical or placebo decompression could potentially unblind post-operative ward staff. Ward staff could inadvertently disclose group allocation to the participant	For participants in the placebo group, the operating team delays closing of the wound for a time period equivalent to the theatre time required for routine lumbar decompression surgeries
**Medical records:** Ward staff could become unblinded if group allocation is documented in the participant’s medical notes	A standardised trial-specific operation report, which documents that a SUcceSS trial procedure has been administered but without disclosure of group allocation, is inserted into the participant’s medical records

**Table 2:** Potential sources of accidental unblinding and risk mitigation strategies: post-operative phase

Potential source of accidental unblinding	Risk mitigation strategy
**Interactions between the unblinded operating team and blinded post-operative staff or trial participants:** The unblinded operating team could inadvertently disclose treatment allocation to the post-operative ward staff, or even the participant during routine standard inpatient and outpatient post-operative clinical reviews	Firstly, only essential personnel who are critical to safe and successful randomisation are allowed to be present during the operation. In SUcceSS, the operating team typically consists of the operating surgeon, theatre nurse/s, the anaesthetist, and occasionally a surgical trainee. After randomisation (i.e. operation), except for the unblinded operating surgeon who will only interact with the blinded participant or post-operative ward staff under explicit circumstances, all other members of the operating team do not have any further interactions with the trial participant or post-operative ward staff To limit contact between the unblinded operating team and post-operative ward staff, as well as trial participants, all participants are assigned a blinded post-operative surgeon. The blinded surgeon is trained in the trial protocol and is responsible for performing all post-operative inpatient consultations, reviewing medical records for any adverse events experienced during the hospital stay, and performing all routine standard post-operative outpatient consultations. Post-operatively, the blinded post-operative surgeon will only consult with the unblinded operating surgeon if the blinded post-operative surgeon is concerned that the participant may require further surgery. A participant will only interact with the unblinded operating surgeon if further surgery is indicated. Even if further surgery is indicated, the participant is encouraged to remain blinded to allocation, as revision surgery is a common complication of lumbar decompression surgery
**Interactions between unblinded theatre staff and blinded post-operative staff:** Theatre staff who are present during randomisation (surgery) may inadvertently disclose trial allocation to post-operative ward staff	When new recruitment sites are implemented, training sessions are scheduled with all theatre staff (e.g. operating surgeons and trainees, theatre nurses, anaesthetists) to ensure that staff are aware group allocation should not be discussed outside of the operating theatre. Refresher training sessions are also scheduled periodically (i.e. before new participants undergo randomisation)
**Billing:** In Australia, standard practice for private health insurers and the universal health care system (Medicare) is to send medical invoices, which include details of the procedures performed, directly to patients. Upon receipt, participants could accidentally become unblinded. In the SUcceSS trial, medical invoices may be related to the surgery or related investigations (e.g. blood test, imaging)	Prior to commencement of recruitment at each site, a site-specific billing protocol is established with the administration and finance departments of the partnering hospitals. Billing protocols are also established with private health insurers. The protocols ensure that medical records are kept blinded, and invoices are sent directly to the unblinded central clinical trial co-ordinator instead of the participant
**Investigational images and reports:** Post-operatively, the blinded surgeon, GP, or other clinicians implicated in the participant’s care may order investigations such as lumbar x-rays or MRIs. It is possible that images or reports could unblind the clinician, and/or inadvertently the participant, to group allocation	Prior to participation, participants are encouraged to discuss their involvement in the trial with their GP. If necessary, before the index surgery, the GP is alerted to the importance of maintaining participant blinding during the trial. After participants are discharged from hospital, a standardised letter is sent to their GP requesting that lumbar imaging should be avoided until completion of the 24-month follow-up, or, at least after the 3-month follow-up (i.e. primary endpoint). If further imaging is deemed necessary, the trial operations team will contact the clinician who has requested further imaging and remind them that images and reports should not be sent to the participant, and group allocation should not be revealed to the participant without approval from the chief investigator. Where feasible, the trial operations team aims to ensure that the unblinded surgeon is the only personnel with access to images or reports

*GP* general practitioner

Understanding the nuances of the surgical procedure under investigation, such as the routine clinical pathway or prognosis of patients after surgery, is also beneficial for pinpointing and mitigating opportunities for accidental unblinding and/or high rates of treatment crossovers. In the context of the SUcceSS trial, there is evidence suggesting that lumbar decompression surgery is associated with a revision rate of 1 in 10 within 4 years [16]. Therefore, at the point of trial entry, all participants are informed that (i) revision surgery is a common complication of lumbar decompression surgery; (ii) further surgery may be required within both arms of the trial; (iii) consequently, undergoing further surgery does not insinuate group allocation. If further surgery is indicated, participants randomised to the placebo surgery group will crossover and receive lumbar decompression surgery, whilst participants randomised to the lumbar decompression surgery group will receive revision decompression or other surgeries as needed (e.g. spinal fusion). In all scenarios, unless a participant explicitly requests to become unblinded, participants are encouraged to remain blinded even if further surgery is indicated. Furthermore, informed by expert clinical opinion and guided by literature, SUcceSS trial investigators established that the beneficial effects of surgical decompression are likely to be apparent by 3 months, and patients with poor clinical response are unlikely to undergo further spinal imaging or revision surgery prior to this time point [5]. Therefore, 3 months after randomisation was specifically selected as the primary end-point for the trial, to ensure participants remain blinded and are less likely to crossover, before the primary end-point.

Finally, with consideration that exacerbation of symptoms may occur after lumbar decompression surgeries, we also co-designed a clinical escalation pathway with the trial surgeons to prioritise participant safety by ensuring provision of optimal standard care, without interfering with participant blinding. In summary, each trial participant is assigned a blinded post-operative surgeon who performs all routine standard post-operative care (e.g. clinical review at approximately 6 weeks and 3 months after the index procedure). If medical complications occur during the post-operative period, the blinded post-operative surgeon will provide routine appropriate care to the participant. This may involve monitoring of symptoms without intervention, investigational imaging, referral to other non-surgical care (e.g. physiotherapy care, spinal injections), and/or recommendations for further surgery. If the blinded post-operative surgeon becomes aware of group allocation at any point (e.g. after reviewing investigational imaging and reports), the participant will remain blinded. In the event additional surgery is advised and the participant is referred to the operation surgeon for clinical consultation, the participant is kept unaware of treatment allocation if further surgery is recommended.

If unblinding occurs, stringent reporting procedures are followed. Regardless of the mechanism of unblinding (e.g. planned, accidental), details surrounding the unblinding incidence are recorded as a clinical file note in the electronic Trial Master File. Further, a record of all instances of unblinding are provided to the reviewing ethics committee in the annual report. Where possible, the research assistant responsible for collecting follow-up data from the participant, over the phone, will remind the participant not to disclose their group allocation when reporting self-reported outcome measures. If the incident is accidental, further reporting measures are taken as accidental unblinding constitutes a protocol deviation. Firstly, the incident must be reported to the sponsor as soon as the research team becomes aware of the incidence. Subsequently, the sponsor advises if further action is required. Further, the incident will also be recorded on a centralised Protocol Deviation/Violation Log which is stored on the electronic Trial Master file. Importantly, efforts are dedicated towards understanding the exact mechanism of the accidental unblinding and preventative measures are implemented to mitigate future occurrences.

As the implemented strategies to minimise accidental blinding are ongoing and have been largely successful to date, it is difficult to determine which strategies have been most effective. To date, only one participant has been accidentally unblinded to group allocation before the primary endpoint. Therefore, it appears that the practice of selecting an appropriate timepoint for randomisation relative to intervention delivery and understanding the nuances of the surgical procedure under investigation may be beneficial to minimise treatment crossovers (to reduce performance bias) and ultimately, preserve the integrity of the trial.

### Unanticipated challenges: the COVID-19 pandemic as an example

Beyond the design and implementation challenges already described, the COVID-19 pandemic has shown that clinical trial activity can quickly become disrupted by unanticipated factors. Even natural disasters and political unrest have the potential to significantly derail or force premature closure of RCTs, regardless of whether they are open-label or placebo-controlled trials.

During the COVID-19 pandemic, Australia and most countries globally were subjected to large-scale lockdowns in efforts to curb the rapid rate of disease transmission. In New South Wales, Australia, where most of the recruiting hospital sites for the SUcceSS trial are situated, bans were imposed on all non-urgent elective surgeries, and limits were placed on the overall capacity for any elective surgery (non-urgent, semi-urgent, urgent), intermittently between March 2020 to March 2022. Stricter and longer bans were imposed in Victoria, Australia, where two additional recruiting hospital sites are situated. These bans were implemented following an intensive period of training surgical site staff on the trial protocol and the commencement of recruitment — all trial momentum at these sites were halted for a protracted duration. As the SUcceSS trial intervention (lumbar decompression surgery) is considered a non-urgent elective surgery, substantial delays in trial recruitment were experienced between 2020 to 2022.

While little can be done to shield RCTs from extreme circumstances, such as government-mandated lockdowns or bans on elective surgeries, systems can be embedded into the trial protocol to minimise the wider impacts of such events on trial productivity. For example, in the SUcceSS trial, remote data collection methods (e.g. phone calls) were embedded into the original protocol design. Although the initial intent was that phone calls were a more appropriate medium to follow up with older people, particularly after receiving surgery, this strategy allowed us to continue collecting outcome measures even when face-to-face research activity was prohibited by the trial Sponsor and hospital sites during the pandemic. Reactively, after face-to-face research was banned during the COVID-19 pandemic, we also pivoted to remote methods for other study procedures. This included conducting trial information sessions with participants over the phone and introducing online consent via REDCap or postal consent methods, to minimise the need for trial staff and patients to attend high-risk health facilities. Site monitoring visits with local hospital staff, which are essential to ensure that recruitment sites are compliant with the trial protocol and relevant regulations, were also converted to virtual meetings for safety reasons. Nevertheless, researchers cannot pre-plan for all unanticipated events. They must exercise diligence in monitoring trial progress and respond swiftly with well-considered risk mitigation strategies when necessary.

## Discussion

RCTs, and especially placebo-controlled surgical trials, are complex trial designs which are destined to face evolving trial-specific obstacles. This paper provides a comprehensive overview of challenges which have been faced during the implementation of one specific placebo-controlled trial of surgical intervention. We acknowledge that the transferability of the challenges, risk mitigation strategies, and the key lessons learned to other trials of this nature remain uncertain. Nevertheless, we have highlighted numerous considerations and practical strategies which future researchers should consider, or build from, when designing and conducting randomised placebo-controlled trials of surgical interventions. Further, we also recommend that when designing a placebo-controlled trial of surgery, future researchers refer to the ASPIRE guidelines which provides a best practice checklist for RCTs including a placebo surgical control [1]. The guideline recommendations, which were partially informed by the SUcceSS trial, cover several key considerations pertaining to the rationale, design (e.g. placebo fidelity, risk mitigation strategies), conduct, interpretation, and translation of placebo-controlled RCTs of surgical interventions.

Reflecting on the challenges experienced during the implementation of the SUcceSS trial, we have identified several limitations which could be considered when designing future placebo-controlled trials of surgical interventions. In the SUcceSS trial, prior to implementation, a feasibility survey of the target patient population was conducted to ascertain the main barriers towards participation in a placebo-controlled trial of decompression surgery. Findings from the survey informed the contents of the first draft of all written and oral patient communication materials for the trial (e.g. critical information which should be included in the patient-facing materials). Patients (consumers) and clinicians were also invited to provide feedback on the draft materials, which were further refined based on additional comments received. Whilst these steps were valuable for ensuring consideration of patient and clinician perspectives on the trial, this approach focused heavily on consultation as opposed to shared decision-making. With an increasing focus on patient and public involvement in research [20], if it is feasible (e.g. availability of resources, preparation, training, time) [21] future researchers should consider involving patients, carers, clinicians, and members of the public early in the trial design process, and at every decision-making level at varying capacities, to facilitate improved trial co-creation. Several studies elaborate on key principles and strategies to meaningfully increase patient and public involvement in research (e.g. co-creation of consent, blinding, and data collection procedures, as well as patient-facing materials) [21, 22]. Increased patient and public involvement may potentially increase patient willingness to participate in a placebo-controlled surgical trial [23], strengthen the justification for the trial, and persuade clinicians of the importance and value of the trial.

Further, in the SUcceSS trial, when face-to-face visits with the research team are not feasible (e.g. participants living in rural areas, participants who have difficulty with access to transport, during periods where face-to-face research activities were prohibited during the peak of the COVID-19 pandemic), trial information and informed consent sessions are performed via phone call. Phone calls have been chosen as the preferred medium, given the anticipated older age of most trial participants. However, future studies should consider whether the use of videoconferencing may be a suitable alternative, as videoconferencing-supported consent appears to be non-inferior to traditional face-to-face consent [24], it allows researchers to visualise non-verbal cues, and it may also enhance rapport. In turn, this may increase patient confidence and willingness to participate in a placebo-controlled trial of a surgical intervention.

### Key lessons learned

Overall, the key lessons learned from the implementation of the SUcceSS trial are:

1. Researchers should proactively involve key stakeholders early in the trial design phase to ascertain the feasibility of recruitment. This promotes engagement and enablement of clinically appropriate strategies to ensure potential participants are fully informed and reassured about possible risks associated with the proposed placebo intervention. It also helps to minimise risks to participant safety and may increase patient willingness to participate in a placebo-controlled trial of a surgical intervention.
2. Researchers should be aware that additional resources are likely to be required for trials of placebo-controlled surgical interventions which are being investigated for the first time. For example, increased budget may be required to account for staff time to conduct a feasibility study to ascertain clinician and patient perspectives about a proposed trial, network with and recruit potential clinicians willing to support the trial, operationalise procedures designed to enhance participant safety, and liaise with patients and members of the public to co-design the trial. Increased budget may also be required to produce consent video materials.
3. Careful consideration should be given towards selection of an appropriate level of placebo fidelity to optimise the balance between selecting a control group which best accounts for the non-specific effects of the index procedure whilst simultaneously considering participant safety.
4. Selecting the optimal timing of randomisation relative to intervention delivery and understanding the nuances of the surgical procedure under investigation can maximise blinding whilst minimising risks to participant safety and reducing treatment crossovers.

## Conclusion

Placebo-controlled trials are considered the gold standard for evaluating the efficacy of healthcare interventions; however, they can be perceived to be controversial and challenging to conduct for surgical treatments. Using the SUcceSS trial as an example, we discuss key challenges which may arise when conducting a randomised placebo-controlled surgical trial, focusing on issues relating to feasibility, participant safety, trial design, and unanticipated challenges such as the COVID-19 pandemic. We also describe numerous strategies employed to address these challenges and their possible effects on trial implementation. Key lessons learned from the SUcceSS trial include (i) involving key stakeholders early and throughout the trial design phase, (ii) anticipating the need for additional resources (e.g. budget, staff time) to operationalise trials of this nature, (iii) carefully considering the appropriate level of placebo fidelity, timing of randomisation relative to intervention delivery, and nuances of the surgical procedure under investigation. Whilst findings are based on one example of a placebo-controlled surgical trial, researchers may benefit from employing or building from the strategies described and lessons learned when designing or implementing future trials of this nature.

## Data Availability

Not applicable.
